# Hygiene performance rating—An auditing scheme for evaluation of slaughter hygiene

**DOI:** 10.1016/j.mex.2020.100829

**Published:** 2020-02-20

**Authors:** O.J. Røtterud, G.E.N. Gravning, S.J. Hauge, O. Alvseike

**Affiliations:** Animalia – Norwegian Meat & Poultry Research Centre, P. O. Box 396 Økern, 0513 Oslo, Norway

**Keywords:** Assessment, Hygiene criteria, Auditing, Slaughter line, Sheep, Cattle

## Abstract

The Hygiene Performance Rating scheme is developed by Animalia in Norway. This unique auditing tool for assessment of slaughter hygiene has been used in Norwegian abattoirs for the last 10 years. The Hygiene Performance Rating scheme visually evaluates and documents each operation on the slaughter line, assessing the factors that can affect the slaughter hygiene. The protocol is based on a systematic evaluation of general hygienic practices of each operation, such as the operators’ hygienic behavior and risk handling of the carcasses, along with routines and management. The scores are registered in a web-based application.

The observations are given a score from 1 to 3, where 1 means “acceptable”, 2 = “potential for improvement”, and 3 = “not acceptable”. Scores for each position is multiplied with a weight factor for hygienic impact and risk (1, 3, 6 or 12) and economic consequences (1 or 2) describing whether the necessary improvement depends on a significant investment (1) or if it is a cheap quick-fix (2) and calculated into a percentage where 100% is perfect hygiene. A presentation of results for the involved parties, including operators, is a crucial part of the implementation of the Hygiene Performance Rating scheme.

• Systematic auditing tool for evaluating slaughter hygiene.
• Investigate and improve slaughter techniques and routines.
• Comprehensive approach to achieve satisfactory results for slaughter hygiene.

## Graphical abstract

**Figure:**
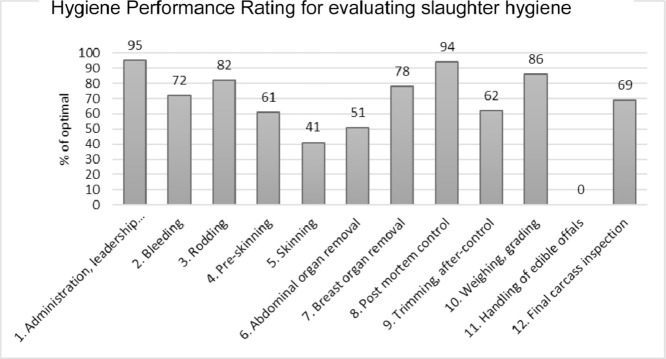

**Table:** Specification table

Subject Area:	*Veterinary Science and Veterinary Medicine*
More specific subject area:	*Slaughter hygiene, auditing, assessment*
Method name:	*Hygiene Performance Rating (HPR)*
Name and reference of original method:	*Not relevant*
Resource availability:	Companies evaluated by the HPR have access to their accounts through a database available from www.animalia.no. The online *HPR database* provides a platform for auditors’ registration of observations, and presentation of the resulting audits.

## Methodology

The different aspects of the Hygiene Performance Rating (HPR) protocol are described in the coming subchapters.

### The hygienic performance rating spreadsheet model

Overall, the HPR addresses factors which have impact on hygienic performance of the slaughter process. These factors include *assessment of premises, assessment of start-up procedures, assessment of operators’ and meat inspectors'compliance with General Hygiene Practise (GHP), among others.*

The HPR protocol is presented in a spreadsheet by Røtterud & Gravning (2019) in supplementary materials [7] and consists of twelve chapters addressing the slaughter procedure for a sheep slaughter line. Each chapter includes a different number of control points (questions). The control points included in the HPR spreadsheet can be relevant for several chapters, hence a crosstabulation occur with same questions being relevant for positions in different chapters.

**Fig. 1:**
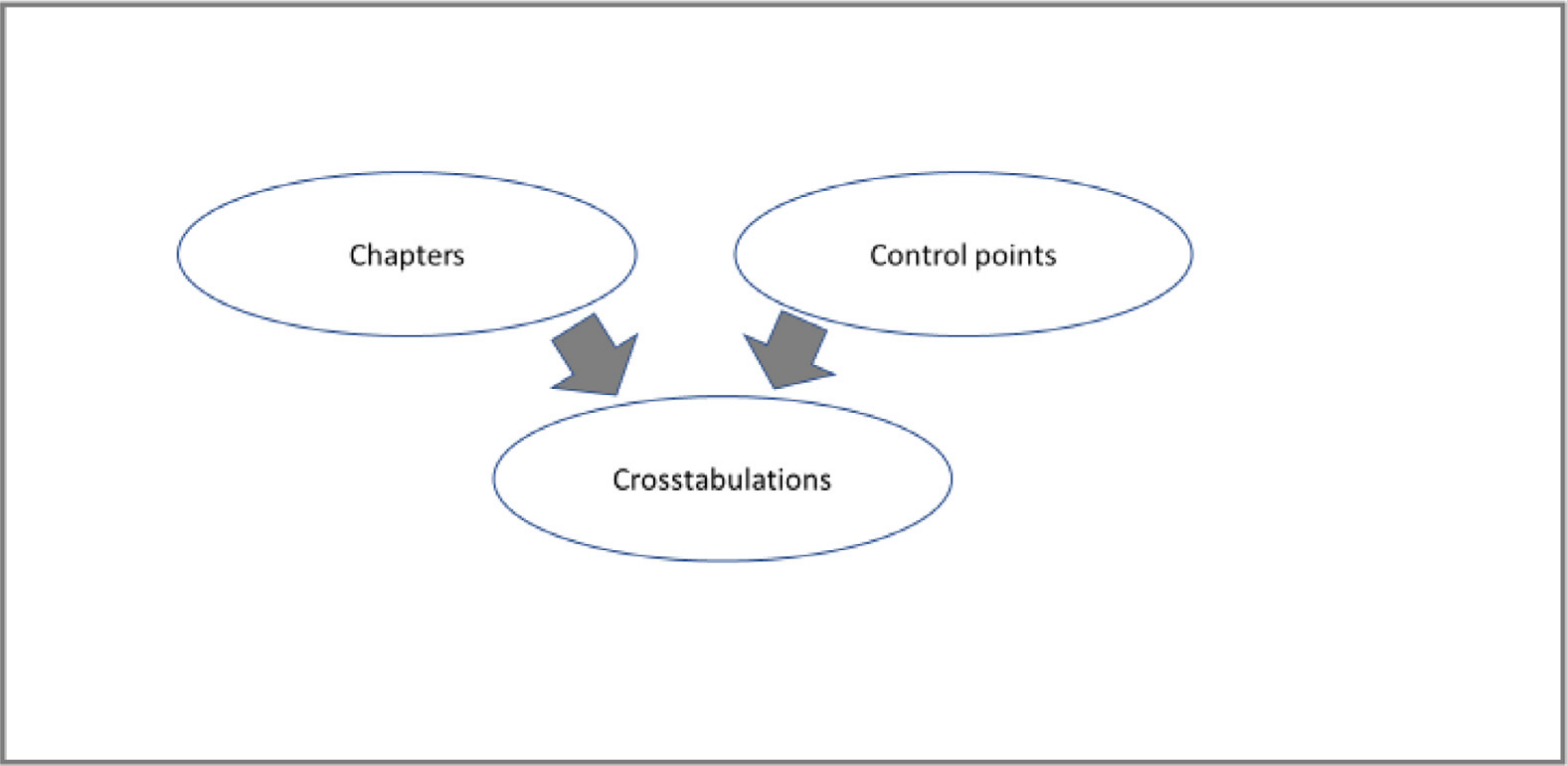
Structure of the hygienic performance rating protocol. Chapters represent predefined positions on the slaughter line. Control points are valid for several chapters, hence a crosstabulation represent the combination of chapters and control points.

An illustration of the overall structure of the HPR protocol is presented in Fig. 1.

#### Chapters in HPR protocol

The HPR protocol has an overall structure divided into chapters. These chapters represent positions along the slaughter line or other factors judged as influencing the slaughter hygiene. In the presented HPR protocol, these chapters are:

(1) Administration, leadership and premises;
(2) Bleeding;
(3) Rodding;
(4) Pre-skinning;
(5) Skinning;
(6) Abdominal organ removal (evisceration);
(7) Breast organ removal (evisceration);
(8) Post mortem control;
(9) Trimming, after-control;
(10) Weighing, grading;
(11) Handling of edible offal;
(12) Final inspection of carcasses.

The outline of the HPR protocol must reflect the process, meaning that the construction of the slaughter line and the organization of the slaughter process is rendered in the protocol.

##### Chapter 1

*Administration, leadership and premises* include factors independent of the position on the slaughter line but regarded important as to influence the slaughter hygiene. It includes construction and type and quality of material of floor, wall and ceiling, pre-slaughter cleaning of facilities, hygienic construction of slaughter-line, presence of leaders in the slaughter hall, communication between operators, among others.

##### Chapter 2–11

The successive chapters 2–11 are named according to the operation on the slaughter line. Several control points (questions) are relevant for more chapters. Not all chapters are relevant for all slaughter lines (e.g., rodding is not always an operation on a slaughter line), and the order of operations may vary. The number of chapters in the HPR protocol can be adjusted to the local organization of operations, and function as template for developing a HPR protocol for auditing slaughter hygiene.

##### Chapter 12

In chapter *12, Final inspection of carcasses*, the control points specifically address activities as observation of fecal content on carcasses, visible remains of wool and blood on carcasses, among others.

#### Control points (questions) in the HPR protocol

The control points (questions) are formulated to address factors that could harm the carcass hygiene. In this HPR protocol, a total of 127 control points are presented in spreadsheet ([7] - HPR, Control points – questions and Crosstabulations). The number of control points or questions in the HPR protocol will vary according to the slaughter line under investigation.

#### Crosstabulation in the HPR protocol

The control points (questions) are repeated in several chapters according to its need. The crosstabulation in the HPR protocol is presented in spreadsheet ([7] - HPR, Control points – questions and Crosstabulations).

### Weighing and calculation of scores

Each chapter along the slaughter line has its fixed set of control points or questions that should be addressed. Not all questions are relevant for all slaughter lines (e.g., rodding), but the HPR protocol takes this matter into consideration so it will influence the result to a minor degree. The control points or questions have a predefined range of what is acceptable, improvements necessary and not acceptable. All observations are scored 1, 2 or 3, where 1 = “acceptable”, 2 = “potential for improvements”, and 3 = “not acceptable”.

The registered values are automatically weighted for pre-set hygienic impact, and then for economic consequences. Weighting for hygienic impact and assumed risk are either 1, 3, 6 or 12. Economic consequences are either weighted 1 or 2, where “1” indicates the abattoir must make a considerable investment, and “2” indicates that a cheaper solution could be implemented. Therefore, the protocol punishes more if it is considered easy or cheap to provide technical solutions that will increase likelihood for the operators to perform their tasks in compliance with GHP.

The calculations of HPR score and total score with economic and hygienic weighing is presented in Table 1. The weighted result is the *score of each control point times the economic and hygienic weighing.* The so-called “theoretical maximum score” is the highest score possible for a control point (=3) times the economic and hygienic weighing.

**Table 1:** Calculation of score and totals score with economic and hygienic weighing.

*Calculations:*
Weighted result = score of observation x economic weighing x hygienic weighing
Theoretical maximum = 3 (score of observation) x economic weighing x hygienic weighing
If observation = 0 – then both weighted result and theoretical maximum = 0.
If observation = 1 – weighted result is set to 0.
If observation is 2 or 3 - weighted result is calculated as described.
*Calculation of single score in% =* 100 *- (weighted result / theoretical maximum) x* 100
*Calculation of total score in% =* 100 *- (sum of weighted result for each chapter / sum of theoretical maximum for all chapters) x* 100
Results for total score level:
A: Total score 85,1 - 100,0% (No chapters < 60,0%)
B: Total score 70,1 - 85,0% (No chapters < 50,0%)
C: Total score 55,1 - 70,0%
D: Total score 0 - 55,0%

If the score of a control point equals “0”, then both the economic and hygienic weighing is also “0”. If score of control point equals “1”, consequently both economic and hygienic weighing is “0”. If score of a control point is “2” or “3”, then the weighted result is calculated as described above.

To get the score result in percentage, the calculation is 100 *minus the weighted result divided by the theoretical maximum times* 100*.*

To get the total score result in percentage, the calculation is 100 *minus total sum of the weighted result for all chapters divided by the total sum of theoretical maximum from all chapters times* 100*.*

A score value is first calculated for each chapter. These scores are relative percentages of full score (100). The profile of these scores from the chapter in the slaughter line it represents, illustrates where the HPR protocol indicate improvements are mostly needed.

These scores are then weighted into the total score. Both the profile of scores and total scores might be applied for benchmarking, but it is underlined that the local management should focus on the direct observations and comments.

**Fig. 2:**
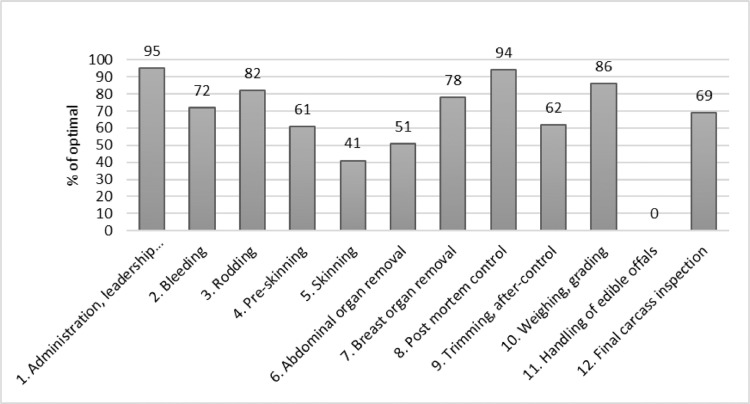
Example on how results from the Hygiene Performance Rating protocol are presented for each position in a sheep slaughter line.

Fig. 2 shows the results from the Hygienic Performance Rating protocol for all positions, 1–12, in a sheep slaughter line. The scores are given as percentage and calculated as described in Table 1.

To ease the impression on how the HPR protocol is filled out, an example of filling out is presented in Table 2. The control points are registered in the skinning position at a sheep slaughter line. The results for the observation, the weighting, theoretical maximum and relevant comments are presented.

**Table 2:** Example of registrations in the Hygienic Performance Rating protocol for the position “skinning” in a sheep slaughter line. The results for observations, the hygienic and economic weighting, the theoretical maximum and comments to the observations are presented. Economic weighting is either valued 1 or 2 and hygienic weighting is valued 1, 3, 6 or 12. See section "Weighing and calculation of scores" for further information on calculations.

Control points	Observed result	Weighting		Weighted result	Theoretical maximum	Comments
		Economic	Hygienic			
All working positions are facilitated with hand wash	2	1	6	12	18	No easy access to hand wash for the operator of the first machine. Operator must use hand wash belonging to neighbouring position
Are knife disinfecting tools available in all working positions?	1	1	6	0	18	Knife is used to a little extent. Last operator on the position uses knife and access to knife disinfecting tool is ok
Are all working positions, where necessary, facilitated with equipment disinfecting tools?		1	6	0	0	
Are sinks situated expediently in accordance with the work flow?	3	1	6	18	18	Hand wash at skinning machine is situated too far from operator
Water temperature in knife disinfecting tool at start up slaughtering	1	2	6	0	36	87,0 °C
Temperature in equipment disinfecting tool at start up slaughtering		2	6	0	0	
Visible contaminations from skin onto carcass	3	2	12	72	72	Frequency 3%

### Implementation of the HPR-protocol

According to the HPR protocol, the assessment starts in the morning, before the slaughtering has started, with an inspection of the quality of cleanliness of the slaughter line, the working tools, observation of the preparation routines of the operators and measuring temperature in knife and equipment disinfection tools, and it finishes when slaughtering ends that same day.

In the HPR protocol, the *worst deviation* observed is registered for operator behaviours, and not the average of the operators, while the *frequency* of errors is used when it comes to indicators as the percentage of rectums and intestines accidentally punctured or the percentage of carcasses with remnants of hide/fleece after deskinning.

Filling out the HPR protocol follows the activity on the slaughter line. For example, information on the quality, that is the appropriateness, thoroughness and use of soap, and frequency of hand washing is collected. Also routines on use and quality of two-knives method with knife disinfection tool [6] and compliance with GHP is registered in the protocol. For all slaughter operations, a major focus is on the contact between the clean carcass and (dirty) hands or knives, equipment, furnishings, among others.

### Feedback and report

A customised presentation is made immediately after completing the HPR protocol, based on observations, measurements, counts, photos and videos. The operators, management and local food authorities are invited to attend when the presentation is given. The results and recommendations are underpinned by photos and videos taken at the slaughter line. The operators see their own behavior. The experience is that the objective registrations are very well accepted, and the best thing is that it stimulates the colleagues to discuss experiences and suggestions how the deviation can be solved. This mobilization of competence seems to be very important to motivate changes and improvements. Also, in many abattoirs, representatives for the authorities (continuously present in the daily operations in abattoirs) and management are normally not confronted for their own habits and practises. Apparently, focus also on these persons contribute to motivation in the whole group.

Simple drawings and animations are used to overcome language problems and simplify the “take home” message as many of the operators speak foreign languages. For each abattoir, a report is provided on-line via the Hygienic Performance Rating Database, described in chapter 1.5, containing detailed results from the HPR assessment, comments and general recommendations, where appropriate.

### Hygienic performance rating database and analytic tool

A database is developed to gather the information from the registration in the HPR protocol. This database is used as the platform for making reports after conducting hygienic audit in abattoirs. The structure of the data in the HPR protocol equals the structure of the data in the database. The final report with scores and comments and graphically presented, are made available on-line for the abattoirs. When planning for a new hygienic audit of an abattoir, it is convenient to use the report in the database as the template for the HPR protocol to be filled out.

## Additional information

The Hazard Analysis Critical Control Points (HACCP) and Good Hygiene Practise (GHP) concepts were introduced in Codex Alimentarius’ standards [2,3] and implemented in European legislation from the 1990′s. Validation is a basic element of HACCP and shall “obtain evidence that the elements of the HACCP plan are effective”. This has been approached by internal and external auditing. Furthermore, verification is “the application of methods, procedures, tests and other evaluations, in addition to monitoring to determine compliance with the HACCP plan” and is also the 6th principle of HACCP. In the meat sector, microbiological sampling is the central objective method for verification of hygiene [4]. A study by Alvseike et al. [1] performed in 20 European slaughter lines evaluated the HPR scheme by comparing with microbiological testing of carcasses slaughtered the same day as the audit was done. A close relationship was found between the total HPR score and the *Enterobacteriaceae* and *E. coli* results of the carcasses. This high correlation suggests that HPR could be a useful proxy measure for improving slaughter hygiene and risk management.

In practise, a technical approach was normally applied where auditors examined the Food Business Operators’ documentation and reported HACCP systematic deviations and non-compliant practises. These reports were typically addressed to the management. A system weakness was observed; the feedback from auditors did not reach the operators or they did not understand the logic and content. Hence, the motivation for improvement was apparently reduced.

Also, the legislation represents the common lowest acceptable condition. To comply with regulation does not necessarily meet the ambitions of every Food Business Operator (FBO), and the horizontal design of European Food legislation provides room for more variation regarding how the objective targets can be achieved. Many FBOs therefore appreciate support to identify their main weaknesses, and then what would be risk-based decisions and investments.

The Hygiene Performance Rating scheme was developed to meet these challenges and is partly based on the framework of the *Ethical Audit* [8]. HPR is developed and adjusted to fit the working stations on a slaughter line but also to include other relevant factors as leadership and management. HPR addresses activities along the slaughter line from stunning and killing to grading of the finished carcasses. HPR is based on a *visual, systematic evaluation of hygienic practices,* performed by one trained external assessor. There is a need of regularly calibrations between the different assessors to keep the assessments as equally as possible. Factors that can affect slaughter hygiene in the operations along the slaughter line are assessed in detail, with focus on the operators’ behavior and handling of the carcasses in addition to the facilitation and features available. A closing meeting with leaders and operators, where findings are presented, is a crucial part of the implementation of HPR. As mentioned above, it is known that the classic auditing approach can have weaknesses when it comes to addressing the findings back to the operators. The closing meeting is a thorough review of observations and findings with pictures or videos illustrating both good and bad practice and routines, securing that the “take home”-message is mediated in a pedagogic and understandable way.

The HPR protocol has been developed since 2002 by Animalia Norwegian Meat and Poultry Research Center, Norway [5]. The judgements are qualitative based on *experience and best practise* and adjusted in iterative manner. The protocol has been iteratively evaluated and adjusted through the last 15 years. HPR-protocols for assessing hygiene in beef, sheep, pork and turkey slaughter lines are available. For this present method description, the sheep accounting protocol is described.

Supplementary material with extensive tables for control points and crosstabulation can be found in the online version

## Declaration of Competing Interest

The authors declare that they have no known competing financial interests or personal relationships that could have appeared to influence the work reported in this paper.

## Appendix Supplementary materials

Image, application 1
